# Multiphoton Imaging of Ca^2+^ Instability in Acute Myocardial Slices from a *RyR2*^R2474S^ Murine Model of Catecholaminergic Polymorphic Ventricular Tachycardia

**DOI:** 10.3390/jcm10132821

**Published:** 2021-06-26

**Authors:** Giulia Borile, Tania Zaglia, Stephan E. Lehnart, Marco Mongillo

**Affiliations:** 1 Department of Biomedical Sciences, University of Padova, Via Ugo Bassi 58/B, 35131 Padova, Italy; giulia.borile@unipd.it (G.B.); tania.zaglia@unipd.it (T.Z.); 2 Veneto Institute of Molecular Medicine, Via Orus 2, 35129 Padova, Italy; 3 Heart Research Heart Research Center Göttingen, Cellular Biophysics and Translational Cardi-Ology Section, Department of Cardiology & Pulmonology, University Medical Center Göttingen, 37073 Göttingen, Germany; slehnart@med.uni-goettingen.de; 4 DZHK (German Centre for Cardiovascular Research), Partner Site Göttingen, 37073 Göttingen, Germany

**Keywords:** catecholaminergic polymorphic ventricular tachycardia, arrhythmias, ryanodine receptor 2, Ca^2+^ imaging, cardiomyocytes, cardiac slices

## Abstract

Catecholaminergic Polymorphic Ventricular Tachycardia (CPVT) is a familial stress-induced arrhythmia syndrome, mostly caused by mutations in Ryanodine receptor 2 (*RyR*2), the sarcoplasmic reticulum (SR) Ca^2+^ release channel in cardiomyocytes. Pathogenetic mutations lead to gain of function in the channel, causing arrhythmias by promoting diastolic spontaneous Ca^2+^ release (SCR) from the SR and delayed afterdepolarizations. While the study of Ca^2+^ dynamics in single cells from murine CPVT models has increased our understanding of the disease pathogenesis, questions remain on the mechanisms triggering the lethal arrhythmias at tissue level. Here, we combined subcellular analysis of Ca^2+^ signals in isolated cardiomyocytes and in acute thick ventricular slices of *RyR2*^R2474S^ knock-in mice, electrically paced at different rates (1–5 Hz), to identify arrhythmogenic Ca^2+^ dynamics, from the sub- to the multicellular perspective. In both models, *RyR2*^R2474S^ cardiomyocytes had increased propensity to develop SCR upon adrenergic stimulation, which manifested, in the slices, with Ca^2+^ alternans and synchronous Ca^2+^ release events in neighboring cardiomyocytes. Analysis of Ca^2+^ dynamics in multiple cells in the tissue suggests that SCRs beget SCRs in contiguous cells, overcoming the protective electrotonic myocardial coupling, and potentially generating arrhythmia triggering foci. We suggest that intercellular interactions may underscore arrhythmic propensity in CPVT hearts with ‘leaky’ RyR2.

## 1. Introduction

Alterations in intracellular Ca^2+^ signaling have been associated with a wide variety of cardiovascular pathologies, ranging from myocardial hypertrophy to heart failure and arrhythmias [1,2,3,4]. In these conditions, dysregulated Ca^2+^ signals cause transcriptional effects (as in hypertrophy), reduced strength of cardiac contraction (as in heart failure) or altered generation and propagation of electric heart activation (as in arrhythmias) [1,2,3,4]. Catecholaminergic Polymorphic Ventricular Tachycardia (CPVT) is one of the most severe inherited arrhythmia syndromes, characteristically leading to stress-induced ventricular arrhythmias and sudden cardiac death (SCD) in children and young adults [5,6,7]. Differently from other genetic forms of cardiac arrhythmias (i.e., Arrhythmogenic Cardiomyopathy) [8,9], CPVT patients typically display hearts with a normal structure [5,6,7]. In the dominant form, CPVT1 is caused by mutations in the *RyR2* gene, encoding the cardiac intracellular Ca^2+^ release channel (ryanodine receptor 2), the main Ca^2+^ release channel of the sarcoplasmic reticulum (SR) [5].

Patients with *RyR2* mutations typically experience sudden arrhythmias during exercise or emotional stresses [5], but may live event-free for decades, even as professional athletes training and competing on a regular basis. This indicates that the development of ventricular arrhythmias initiates when transient mechanisms overcome a protective threshold [10], likely as result of the combination of stress-dependent effects (e.g., activation of sympathetic neurons, SNs) and coincidental factors, not entirely identified. However, specific mechanisms leading to transient electrical hyperexcitability of heart cells, and acting as arrhythmia triggers, have not been identified thus far. It has previously been reported that increased arrhythmic risk correlates with depressed cardiac pump function (ejection fraction), suggesting that cellular signals, activated during contractile dysfunction, may be shared with those contributing to arrhythmia initiation. The most likely candidates are Ca^2+^-dependent effects, which, on the one hand, underlie excitation–contraction coupling [11] and, on the other, increase the likelihood of arrhythmogenic intracellular Ca^2+^ events [7]. Remarkably, mutant RyR2 channels, harboring the CPVT-linked single-point mutation *RyR2*^R2474S^ (*RyR2*^RS^), show increased open probability (Po) when PKA-phosphorylated [7,12,13], which alters local control of Ca^2+^ signals at RyR2 clusters, causing increased diastolic Ca^2+^ leak from the SR in CPVT cardiomyocytes (CMs) challenged with β-adrenergic (β-AR) agonists [7,14]. The resulting Ca^2+^ extrusion via the Na^+^/Ca^2+^ exchanger (NCX) operating in forward mode generates an inward depolarizing current (I_ti_), which leads to delayed afterdepolarizations (DADs), and, if DADs reach the activation threshold of Na^+^ channels, an elicited action potential (AP) causes triggered activity [15]. While this is a well-accepted mechanism causally linking, at the single-cell level, the gain of function of CPVT mutant *RyR2* to transient diastolic SR Ca^2+^ leak and membrane depolarization caused by DADs, how the altered cellular function causes CPVT at the whole-heart and in vivo level is less well understood. In order to function as an effective arrhythmia trigger, DAD-dependent CM depolarizations must occur simultaneously in a sufficient number of CMs to overcome the protective current sink of the electrotonically coupled myocardium [16]. We previously demonstrated that, in the normal mouse heart, this number approximates 2000 contiguous working or 150 conducting CMs [17]. Whether cell- or tissue-dependent factors may promote spatial and temporal synchronization of DADs in the different cells of the intact CPVT heart, thus participating in the generation and sustenance of arrhythmias, is unknown. 

We, thus, aimed to investigate the myocardial dynamics of arrhythmia generation in CPVT, using a previously developed knock-in (KI) *RyR2*^RS^ mouse, and we studied Ca^2+^ dynamics with fluorescence imaging in two different experimental settings: (i) freshly isolated adult CMs, which allow faster imaging rate at elevated spatial resolution [7], and (ii) cardiac slices, which allow analyzing cells in their intact tissue environment, as well as determining whether reciprocal, synergistic influences between neighboring cells may participate in amplifying the arrhythmic effect of the *RyR2* CPVT mutation through direct cell–cell interactions [18,19,20,21,22,23,24,25].

## 2. Materials and Methods

### 2.1. Animal Models

In this study, we used KI mice carrying the human CPVT-linked missense mutation (R2474S) in the *RyR2* gene (*RyR2*^RS/wt^ mice) [7]. Mice, which were kindly provided by Andrew R. Marks, were backcrossed for at least 25 generations into the C57BL/6J background, and wild-type (WT) littermates were used as controls. We here used both young (P2–P21) and adult (3–4 months) male mice. All experimental procedures performed on rodents were approved by the local ethical committee and the Ministry of Health (communication number VIMM/C53, VIMM/C54; 11/2011), in compliance with Italian Animal Welfare Law (Law n 116/1992). All procedures were performed by trained personnel with documented formal training and previous experience in experimental animal handling and care. All procedures were refined prior to starting the study, and the number of animals was calculated to use the least number of animals sufficient to achieve statistical significance according to sample power calculation.

### 2.2. Isolation of Cardiomyocytes from Adult Mouse Hearts

CMs were isolated from 3–4 months old *RyR2*^wt/wt^ (WT) or *RyR2*^RS/wt^ (CPVT) mice, as described in [7]. Cells were plated on laminin-coated coverslips, incubated for 15 min with 2.5 μM Fluo-4-AM, in 0.2% Pluronic F-127 (Invitrogen, Thermo Fisher Scientific, Waltham, MA, USA) and 10 μM sulfynpirazone-containing Tyrode solution, and subsequently washed in dye-free medium for 30 additional min. Cells were, thus, transferred on the microscope stage, which was connected to the field stimulator. Fluorescence Ca^2+^ imaging was performed in Fluo-4 loaded CMs, using the line-scan mode (1 line/1.8 ms) of the confocal laser scanning microscope (Zeiss 5Live, Zeiss GmBH, Carl Zeiss AG, Oberkochen, Germany), with the field paced at 1 Hz. 

### 2.3. Preparation of Acute Heart Slices and Loading with Fluorescent Ca^2+^ Dye

Acute ventricular heart slices were cut following the protocol described in [18]. In detail, young (P2–P21) CPVT and WT mice were sacrificed, and the heart was quickly excised and washed in ice-cold Ca^2+^-free Tyrode solution (136 NaCl; 5.4 KCl; 0.33 NaH_2_PO_4_; 1 MgCl_2_; 10 glucose; 5 HEPES, in mM; pH 7.40 with NaOH). Atria were excised, while ventricles were embedded in 4% Low-Melting Agarose (SIGMA-Aldrich, Burlington, VT, USA) in Tyrode solution at 35 °C, and slowly cooled at room temperature. Once the Agarose cast solidified, 450 µm thick transversal slices were cut in ice-cold Ca^2+^-free Tyrode solution with a vibratome (Leica GmBH, Leica Camera AG, Wetzlar, Germany), left to equilibrate in 1 mM CaCl_2_ Tyrode at room temperature for 5 min, and subsequently maintained into Recovery Medium (DMEM/F12, supplemented with 20% Knock-out Replacement Serum, Invitrogen, Thermo Fisher Scientific, Waltham, MA, USA) [26] in a humidified atmosphere, with 5% CO_2_ at 37 °C for at least 1 h before loading. Slices were perfused on both sides to minimize hypoxic damage. After recovery, slices were loaded with the Ca^2+^-sensitive indicator Fluo-4-AM (5 µM, Invitrogen, Thermo Fisher Scientific, Waltham, MA, USA) for 40 min at 37 °C in 2 mM Ca^2+^ Tyrode solution, supplemented with 0.2% Pluronic F-127 (Invitrogen) and 10 µM sulfinpyrazone (SIGMA-Aldrich, Burlington, VT, USA) in a 12-well plate positioned on a shaker.

### 2.4. Two-Photon Emission Microscope

A multi-photon system (Radiance 2100 MP; Bio-Rad Laboratories, Watford, UK), equipped with a Tsunami mode-locked, tunable, femtosecond-pulsed Ti/sapphire laser (Ti:Sa), optically pumped by a Millennia VsS 5W green laser (Spectra Physics, MKS Instruments, Inc., Andover, MA, USA), was used for two-photon microscopy (see paragraph 2.5). The laser output generates 100 fs pulse trains at a rate of 82 MHz. The excitation wavelength was set at 785 nm and controlled with a spectrometer (Ocean Optics USB2000; Duiven, The Netherlands). The microscope (Eclipse E600FN; Nikon, Tokyo, Japan) was equipped with a water immersion objective (40×/0.8 W, Nikon, Tokyo, Japan) and a non-descanned detection system (Direct Detection System, Bio-Rad, Hercules, CA, USA), fitted with a 500LP DC dichroic mirror and HQ535/50 emission filter (Chroma Technology Corp, Bellows Falls, VT, USA), which were used in the emission path for the detection of emitted fluorescence. Data were acquired using the proprietary software LaserSharp2000, (Bio-Rad, Hercules, CA, USA) and analyzed with Fiji *(“Fiji: an open-source platform for biological-image analysis”, Nature methods 9(7): 676–682*).

### 2.5. Two-Photon Ca^2+^ Imaging on Heart Slices

Viable heart slices, loaded with Fluo-4-AM, were transferred on a custom-made perfusion chamber on the stage of the two-photon microscope and perfused with a 2 mM Ca^2+^ oxygenated Tyrode solution, supplemented with 10 µM sulfinpyrazone to avoid dye extrusion. To prevent curling and movement, slices were held down through a homemade platinum holder. Slices were field-stimulated by applying brief voltage square waveforms (5 ms) directly under the microscope with a voltage difference at the slice sides of 5 V/cm. To minimize the risk of hypoxia during the experiments, we used short pacing protocols at room temperature, as suggested by Barclay with respect to a similar cardiac tissue preparation [27]. Images were, thus, collected upon excitation of the sample with 785 nm excitation wavelength and collection occurred through a bandpass filter selecting the green emission (535 ± 50 nm). Images were recorded by the microscope software and stored. Changes in Ca^2+^ concentration were observed as variations in the green emission of the sample upon excitation. All experiments were performed at room temperature. 

### 2.6. Image Analysis

ImageJ (Wayne Rasband, NIH, Bethesda, ZeniMax Media, Rockville, MD, USA) and Clampfit 10.0 (pClamp suite, Molecular Devices) were used as the main software packages for image analysis. The fluorescence emitted by the slices was expressed as F/F0, where F represents the fluorescence at a time t and F0 the minimum fluorescence value of the same cell. All fitting procedures were made with Clampfit 10.0. The amplitude of Ca^2+^ release events included in the analysis was at least 10% that of the triggered Ca^2+^ transients, whereas Ca^2+^ alternans were defined when two consecutive Ca^2+^ transient amplitudes differed by at least 10%, and this alternating behavior was maintained for at least 10 transients.

### 2.7. Statistical Analysis

The sample size per group was estimated with sample power analysis using previously determined standard deviations in similar experiments. Data were analyzed using Prism Software (GraphPad, La Jolla, CA, USA). For normally distributed experimental variables, determined with Shapiro–Wilk normality test, data were expressed as mean ± SD (standard deviation), and difference among groups was determined by unpaired *t*-test, ANOVA, and Welch’s unequal variance unpaired test. Differences with a *p*-value lower than 0.05 were considered significant.

## 3. Results

### 3.1. Diastolic Sarcoplasmic Reticulum Ca^2+^ Leak in Isolated RyR2^RS/wt^ CPVT Cardiomyocytes

**Figure 1:**
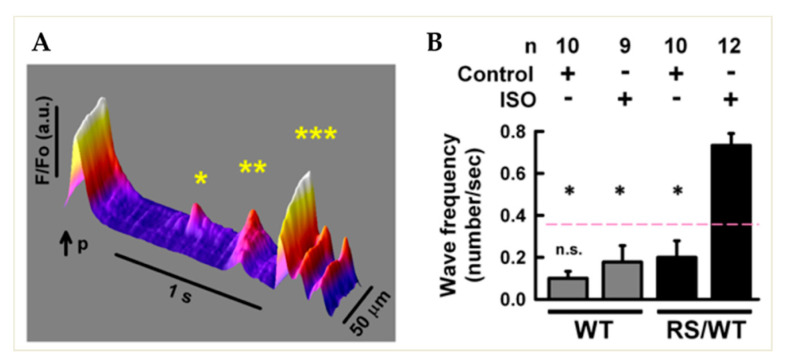
Spontaneous Ca^2+^ release in adult cardiomyocytes isolated from CPVT mouse hearts. *(***A**) Fluorescence image of Ca^2+^ dynamics monitored with confocal microscopy in a heterozygous *RyR2*^RS/wt^ (RS/WT) CM during isoproterenol (ISO, 1 μM) stimulation. Upon steady-state pacing, the last depolarization pulse (p) activates a regular intracellular Ca^2+^ transient, followed by abnormal spontaneous (un-paced) Ca^2+^ waves: * localized; ** cell-wide; *** regenerative. F/Fo, normalized fluorescence Ca^2+^ signal intensity. (**B**) Bar graph summarizing cell-wide Ca^2+^ wave occurrence in WT and CPVT CMs in basal conditions vs. ISO stimulation. Error bars represent SD; * *p* < 0.05.

In accordance with the dominant inheritance of the disease in humans, we investigated Ca^2+^ dynamics in CMs obtained from mice heterozygous for the *RyR2*^R2474S^ point mutation (*RyR2*^RS/wt^), previously shown to develop Ca^2+^-dependent ventricular arrhythmias and SCD when exposed to exercise and pharmacologic stress [7]. Mutation negative littermates were used as controls (*RyR2*^wt/wt^). Consistent with the increased Po of the CPVT-mutant RyR2 when PKA-phosphorylated, *RyR2*^RS/wt^ CMs had larger and more frequent spontaneous Ca^2+^ sparks when treated with β-AR agonists and developed spontaneous diastolic Ca^2+^ release events [7]. We here extended the analysis and quantitated the number and spatial dynamics of intracellular Ca^2+^ waves in CMs, electrically paced with field stimulation and challenged with the adrenergic agonist, isoproterenol. At the end of the pacing-evoked intracellular Ca^2+^ transient, while cytoplasmic [Ca^2+^] declined for interruption of L-type Ca^2+^ channel input and SR re-uptake, CPVT CMs developed spontaneous Ca^2+^ elevations, which frequently initiated as local events (sparks, marked by * in Figure 1A), spreading and transforming into self-sustained macro-sparks (marked by ** in Figure 1A) and ultimately cell-wide and self-propagating waves (marked by *** in Figure 1A). Consistent with the hypothesis that these events depended on the combinatorial effect of PKA activation on SR Ca^2+^ loading, as well as on the gain of function of phosphorylated mutant RyR2, these spontaneous waves were never observed in control CMs, which only rarely showed macro-sparks (Figure 1B) and were almost never detected in the absence of β-AR stimulation.

### 3.2. Preparation of Acute Myocardial Slices Suited for Ca^2+^ Imaging in Multicellular Heart Tissue

While the study of isolated CMs has allowed refined determination of local and transient Ca^2+^ dynamics [1,2,3,4], single-cell behavior may differ substantially from that of the intact myocardium, in which cell–cell coupling and tissue geometry contribute to shaping Ca^2+^ signals. This aspect is particularly important in arrhythmias, which are multicellular phenomena in which Ca^2+^ dysregulation occurs in a highly interdependent fashion in several excitable cells in the tissue. We, therefore, asked whether mechanisms, possibly underlain by intercellular interactions, may engage a sufficient number of contiguous cells to cause the transient appearance of focal arrhythmia triggers. We, thus, aimed to use an experimental model in which living CMs can be investigated in their intact tissue environment using high resolution fluorescence Ca^2+^ imaging. Acute myocardial slices were obtained from *RyR2*^RS/wt^ and control young mice. In detail, ventricular 450 μm thick transverse slices were cut from freshly harvested hearts, a procedure typically yielding 4–5 slices per heart. Initially, Ca^2+^ dynamics were compared in myocardial slices obtained from hearts of different age, ranging from P2 to P21. While no significant changes were observed in the Ca^2+^ transient amplitude, the time constant of decay progressively decreased along with the postnatal cardiac development and was paralleled by increased protein levels of SERCA-2 in myocardial tissue homogenates (Figure S1, Supplementary Materials). Due to the efficient Fluo-4-AM loading and the ease of manipulation, and given that the time constant of decay of Ca^2+^ transients was similar to that observed in adult CMs, additional experiments were performed using P10–P12 mice. To assess the efficacy of our protocol, heart slices were processed with histological (i.e., hematoxylin/eosin) and immunofluorescence staining. Our analyses showed that myocardial slices retained regular cell organization, with intact and morphologically normal cardiac sarcomeres, with the only exception of the cells close to the sectioning surface (Figure S2, Supplementary Materials). Staining of slices with DAPI and propidium iodide (PI) showed PI-positive nuclei close to the cutting border (Figure S3, Supplementary Materials), while significantly fewer PI-positive nuclei were found at a depth >50 μm, indicating that myocytes below the surface layers were viable. In support of this, most CMs in the slice accumulated the mitochondrial dye tetramethyl-rhodamine (SIGMA-Aldrich), indicating intact mitochondrial membrane potential (data not shown). All the vitality markers analyzed were principally not different between WT and CPVT heart slices. 

### 3.3. Multiphoton Ca^2+^ Imaging in RyR2^RS/wt^ Acute Myocardial Slices Shows ‘Leaky’ RyR2 Behavior

**Figure 2:**
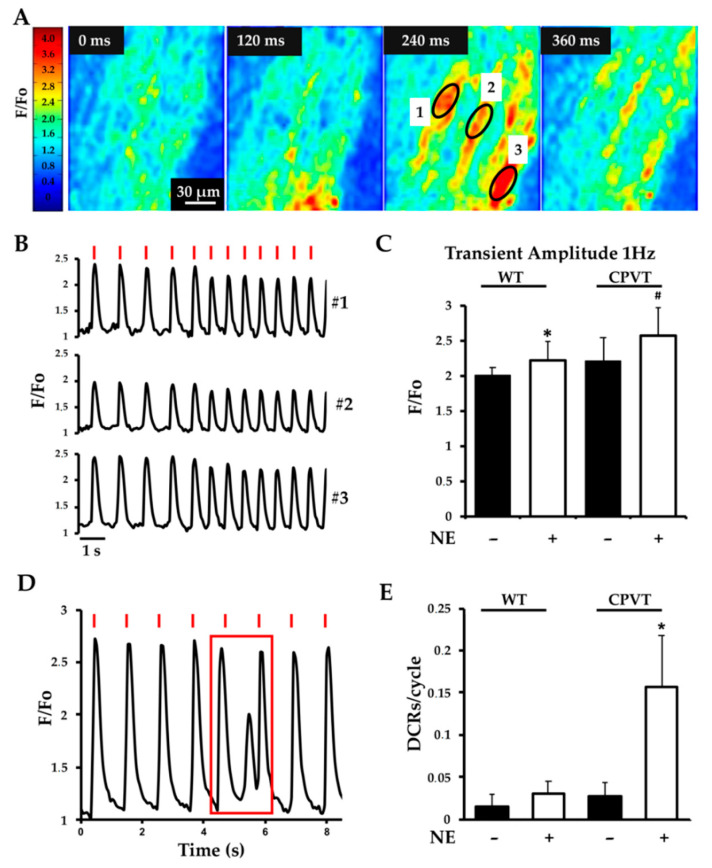
Multiphoton Ca^2+^ imaging of Fluo-4-loaded myocardial slices. (**A**) Time course of Ca^2+^ fluctuations in a heart slice, showing rises in fluorescence intensity, represented in pseudocolor (see reference bar on the left margin of the panel) upon electrical stimulation. F/Fo, normalized fluorescence Ca^2+^ signal intensity. (**B**) Fluorescence intensity profile of three different cells (in **A**, 240 ms) showing synchronized changes during electrical field stimulation. Stimulation pulses are indicated by the red lines. (**C**) Quantification of Ca^2+^ transient amplitude, in both WT and CPVT heart slices, paced at 1 Hz, both in the absence (black bars) and in the presence (white bars) of norepinephrine (NE, 1 μM) (* *p* < 0.05 (WT vs. WT + NE); # *p* < 0.05 (CPVT + NE vs. WT + NE) *n* = 30 cells/condition). (**D**) Representative trace of spontaneous Ca^2+^ release events occurring between two consecutive beats (highlighted by the red box). (**E**) Quantification of SCRs in CPVT and WT slices, in unstimulated conditions and upon perfusion with NE (1 μM). Bars represent SD (* *p* < 0.05 compared to CPVT-NE; *n* = 40 cells for each group).

To image CM Ca^2+^ dynamics, acute heart slices were loaded with the Ca^2+^ dye Fluo-4-AM (Figure S4, Supplementary Materials), subsequently transferred to a field stimulation chamber and bathed in 2 mM Ca^2+^-containing Tyrode solution. Multiphoton microscopy was used to monitor fluorescence changes in cell layers at approximately 100 μm below the slice surface. Electrical pacing resulted in synchronous increases in intracellular Ca^2+^ in the CMs in the imaging field (Figure 2A), as reported in a typical experimental trace shown in (Figure 2B). Ca^2+^ transient amplitude in the individual cells was unchanged between *RyR2*^RS/wt^ and *RyR2*^wt/wt^ myocardium, paced at stimulation rates of 1 Hz (Figure 2B,C) and 2 Hz (not shown). To determine the effect of β-AR activation on intracellular Ca^2+^ dynamics, slices were superfused with 1 μM norepinephrine (NE), during electrical pacing. After 3 min, while the drug diffused into the tissue layers, Ca^2+^ transient amplitude increased (Figure 2C) and, in line with the action of PKA on phospholamban (PLN) phosphorylation and enhanced SERCA-2 activity, the decay kinetics was accelerated, to a similar degree, in control and CPVT hearts. 

In addition, NE stimulation caused the appearance of spontaneous ‘diastolic’ Ca^2+^ release (SCR) events, between two consecutive paced transients (Figure 2D), which were about fourfold more frequent in CMs of CPVT than those of control slices (Figure 2E). These results show that, in the *RyR2*^RS/wt^ myocardium, CMs have increased propensity to generate SCRs upon β-AR stimulation.

**Figure 3:**
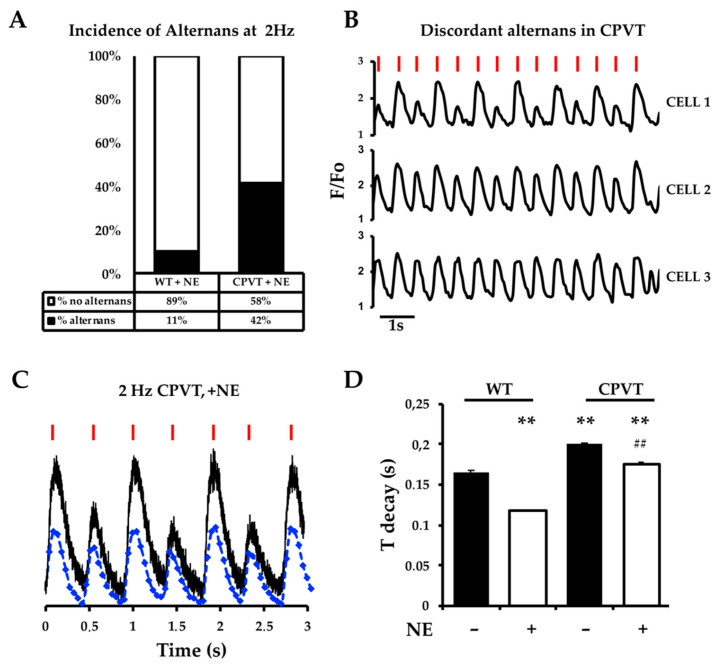
CPVT heart slices develop Ca^2+^ alternans upon β-AR stimulation. (**A**) Fraction of cells developing Ca^2+^ alternans in WT and *RyR2*^RS/wt^ slices, paced at 2 Hz, during β-AR stimulation with NE (1 μM). (**B**) Representative plots of Ca^2+^ oscillations in three adjacent CMs in a *RyR2*^RS/wt^ slice upon NE perfusion, showing alternans synchronous in phase, but not in amplitude (red bars indicate electrical pacing). (**C**) Line scan profiles from CPVT CMs, in basal (blue line) or NE-stimulated (black line) conditions, during 2 Hz stimulation. (**D**) Decay time of Ca^2+^ transients calculated on the line scan profiles in (**C**), for both WT and CPVT slices, paced at 2 Hz. Error bars represent SD (*n* = 25 cells/condition; ** *p* < 0.01 compared to WT; ## *p* < 0.01 compared to CPVT in basal condition).

When the pacing rate was increased to 2 Hz, predominantly in the *RyR2*^RS/wt^ heart slices compared to controls, the amplitude of Ca^2+^ transients alternated on a beat-to-beat basis in a significant number of cells in the imaging field (CPVT: 44 (11/25) vs. WT: 12 (3/24), in %; 6 independent preparations each) (Figure 3A). Ca^2+^ alternans observed during pacing at 2 Hz were synchronous in phase, but not in amplitude, as shown by the fluorescence/time profile of three neighboring cells in Figure 3B, and, in agreement with previous reports [28,29], the decay kinetics of large Ca^2+^ transients tended to be slower than that of small transients (Figure 3C,D), although it fell short of statistical significance (*p* = 0.06). 

### 3.4. Pace-Stop Protocol Uncovers Spontaneous Diastolic Ca^2+^ Leak in CPVT Slices

**Figure 4:**
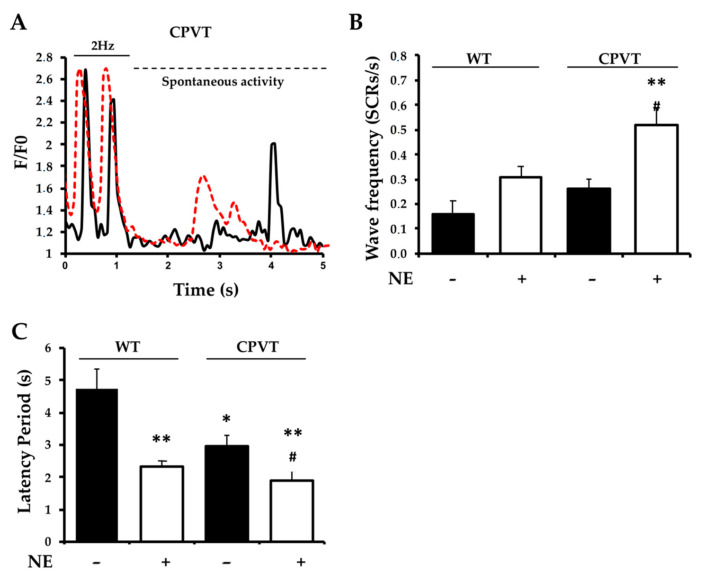
Pace-stop stimulation protocol unmasks spontaneous untriggered Ca^2+^ release in CPVT slices. (**A**) Fluorescence profiles of CMs within cardiac slice from heterozygous *RyR2*^RS/wt^ mice, in control conditions (black trace) and during NE administration (red trace), showing a representative trace of a SCR. (**B**) Quantification of SCRs in the slices from WT and CPVT hearts at baseline and in the presence of NE. Error bars represent SD (** *p* < 0.01 compared to WT *plus* NE; # *p* < 0.001 compared to CPVT in basal condition). (**C**) WT and CPVT slices showed significantly different latency time before the first spontaneous Ca^2+^ release event. Error bars represent SD (* *p* < 0.05 compared to WT; ** *p* < 0.01 compared to WT; # *p* < 0.05 CPVT in basal condition vs. NE administration).

Given the mechanistic link between altered single-channel properties of CPVT mutant *RyR2*, we focused on the investigation of aberrant diastolic Ca^2+^ release from the intracellular stores. To this aim, we used a pace-stop protocol in which, after a brief period (5–10 s) of electrical pacing at 2 Hz, pacing was paused, and [Ca^2+^] declined to levels of unpaced cells (Figure 4A). After a latency significantly shorter in *RyR2*^RS/wt^ than in WT hearts, SCRs occurred in the form of Ca^2+^ macro-sparks and waves [30,31]. In keeping with the results obtained in freshly isolated CMs (see Figure 1), the average number of SCRs in unstimulated slices was modest in CPVT and WT samples, while it was significantly increased upon NE stimulation in CPVT cells (Figure 4B). In slices from *RyR2*^RS/wt^ hearts, the latency to develop Ca^2+^ waves was significantly shorter than in controls (CPVT: 3.0 ± 0.3 vs. WT: 4.7 ± 0.6, in seconds; *n* = 20 (CPVT) and 25 (WT) cells; *p* < 0.05), and the number of cells within the slice showing aberrant activity was markedly increased (CPVT: 39% vs. WT: 15%; 40 cells/genotype). NE (1 μM) administration decreased the latent period to the generation of SCR in both CPVT and control hearts (Figure 4C). The initial Ca^2+^ release generated traveling waves which diffused throughout the whole cell at higher speed in CPVT CMs (CPVT: 57 ± 1 vs. WT: 44 ± 1 in μm/s; *n* = 7 cells for each group, *p* < 0.01), possibly reflecting the increased open probability of *RyR2*^RS/wt^ channels upon phosphorylation.

### 3.5. Intercellular Trasmission of Ca^2+^ Instability in Spatially Defined Myocardial Cell Clusters through Enhancement of SCR Vulnerability in Neighboring Cells

**Figure 5:**
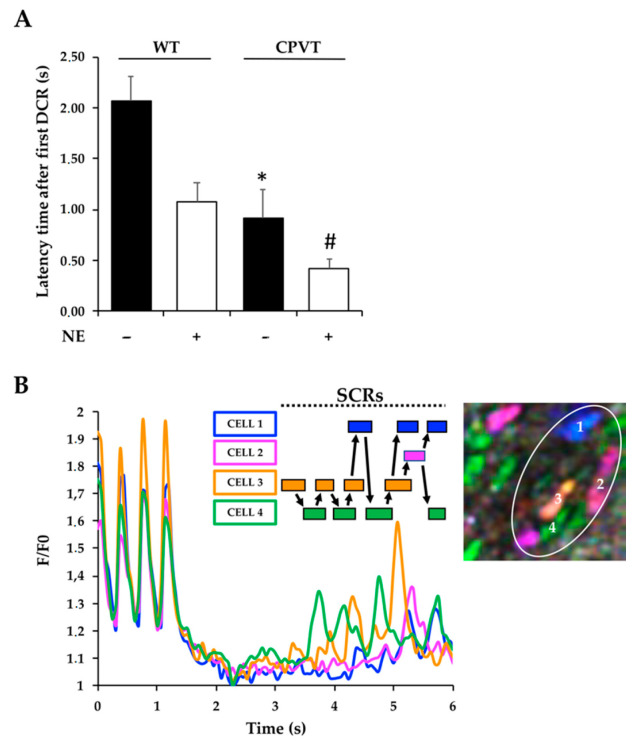
Diastolic SCR synchronization in myocardial cell clusters. (**A**) Cells in CPVT slices showed significantly shorter latency, compared to controls (WT), between the first SCR upon pacing interruption and the subsequent ones in neighboring CMs. Error bars represent SD (* *p* < 0.05 compared to WT in basal condition; # *p* < 0.01 compared to CPVT upon NE stimulation). (**B**) Representative traces of Ca^2+^ dynamics in a CPVT slice that underwent a pace-stop protocol. The colored traces show intracellular Ca^2+^ changes in the cells identified by the same color in the regions of interest (ROIs) delineated in the corresponding image on the right side of the panel. The rectangles above the chart highlight, in the same color coding, the duration of spontaneous Ca^2+^ elevations, defined as increase of the fluorescence signal higher than two SDs of the basal fluorescence intensity. Arrows connect the individual boxes, each one representing a single cell in the field, showing the intercellular transmission of temporally overlapped SCRs in adjacent cells, which underscores the influence SCR may have on further SCRs. In the representative example shown, the initiating event occurs in cell #3 (orange line and box) and is subsequently transmitted to the others.

**Figure 6:**
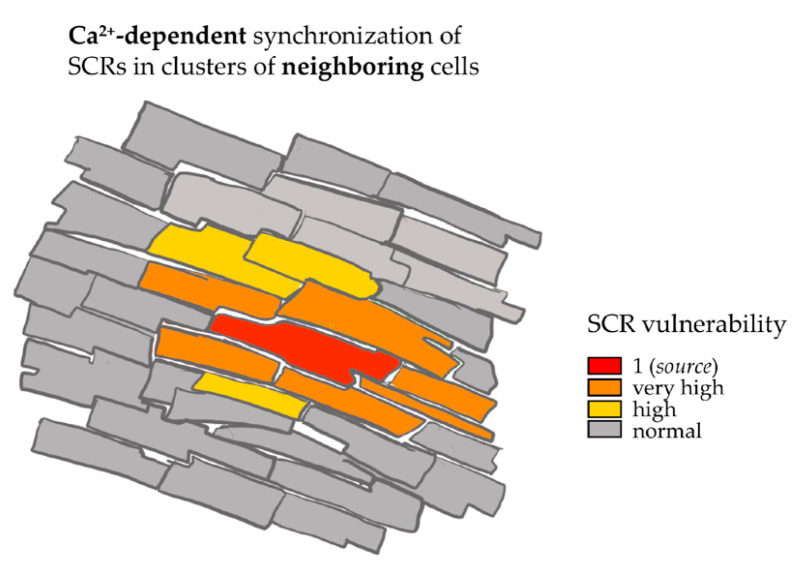
Factors potentially contributing to synchronization of diastolic depolarization in the myocardium. A “source–sink–source” model, showing the effect of a SCR occurring in one myocardial excitable cell (red labeled “source” cell) on vulnerability of surrounding CM to develop, in turn, diastolic “SR leak” and SCR. This positive feedback loop may, thus, “regenerate” a current source, enlarging the area of CMs depolarizing synchronously.

When SCRs were analyzed in all cells in the field of view, it emerged that they tended to occur almost synchronously in neighboring cells. We speculated that, if SCRs were independent in each cell, they would occur with stochastic distribution in any CM in the slice. On the contrary, if reciprocal influence of SCRs existed between coupled cells, we would expect the probability to develop Ca^2+^ release to be higher in cells contacting a “*source*” CM, “*sinking*” the Ca^2+^-dependent depolarizing current (i.e., SCR-dependent I_ti_) at the expense of a slight loss of membrane polarity. However, within the average duration of the transient diastolic Ca^2+^ elevation, occurring in a given cell (CPVT: 915 ± 70 vs. WT: 855 ± 43, in ms; *n* = 40 cells per genotype in six biological replicates; *p* = ns), the probability that a neighboring one would develop, in turn, secondary diastolic Ca^2+^ leak was >80%, much higher than the calculated probability of two independent events with the typical frequency of 0.4/s (average SCR/s/cell for any given cell in the slice) to occur. To further analyze such behavior, we calculated the average latency time, after pacing cessation evoked the first Ca^2+^ release in one of the cells in the field of view, for neighboring cells to develop secondary SCR events. As shown in (Figure 5A), such value was significantly shorter in NE-treated CPVT slices than in all other conditions (CPVT slices in basal conditions, WT slices in either the presence or the absence of NE, *n* = 16 cells per group). This data suggests that reciprocal influence between neighboring CMs might contribute to the synchronization of diastolic Ca^2+^ release events within a cluster of neighboring cells in the same tissue region, as shown in the representative plot of Ca^2+^ fluctuations in regions of interest, corresponding to different cells in the field of view (Figure 5B). This mechanism may suffice to overcome the protective myocardial source–sink mismatch and contribute to generate ectopic arrhythmic foci, supporting the purported model illustrated in Figure 6. 

## 4. Discussion

CPVT is one of the most dramatic stress-dependent genetic arrhythmogenic syndromes, and its dominant inherited variant is caused by various missense mutations in the *RyR2* gene, encoding the main intracellular Ca^2+^ release channel. The disease is frequently the cause of sudden cardiac death (SCD) in young adolescents and athletes. It is well accepted that alterations in intracellular Ca^2+^ handling, due to functional abnormalities of mutant RyR2 channels, have a role in triggering arrhythmias in CPVT hearts, and Ca^2+^-dependent delayed after-depolarizations (DAD) have consistently and causally been linked to triggering arrhythmias in various murine models, harboring different CPVT-linked mutations [7,31,32,33,34]. Although the mechanisms of Ca^2+^-dependent ‘triggered activity’ have been deeply investigated at the cellular level (i.e., in isolated CMs), the relationship between abnormal CM Ca^2+^ cycling and focal excitation at the tissue level remains poorly understood [35,36]. 

A key unresolved question is how spontaneous Ca^2+^ release in individual CMs can cause a sufficiently large membrane depolarization to elicit an ectopic event across the whole myocardium, given that a local depolarizing current is expected to be absorbed by the electrically connected neighboring CMs serving as current sinks [16]. In the last few years, attention has been given toward the clarification of this aspect, and several groups have addressed this point with different experimental approaches, ranging from *in silico* models, isolated CMs, and trabeculae preparations to whole perfused hearts, but a clear identification of the mechanism is still lacking [15,31,33,37]. Given the impact of the arrhythmic consequences of CPVT and the incomplete effectiveness of the conventional pharmacologic therapies, increasing our understanding on how arrhythmias are initiated in the CPVT heart is necessary to identify alternative and effective therapeutic strategies. Here, we used multiphoton microscopy in a tissue model of acute thick cardiac slices to investigate Ca^2+^ dynamics in the functionally intact myocardium of knock-in mice harboring the CPVT mutation *RyR2*^R2474S^.

Murine ventricular slices have been used to monitor electrophysiological parameters and as a promising model for pharmacological drug testing, usually without single-cell resolution [18,19,20,21,22,23,24,25]. We previously validated our slice model and multiphoton fluorescence Ca^2+^ imaging, achieving subcellular resolution and fast sampling rates in myocardial slices from normal WT mice [38]. Other groups have used cardiac slices to study arrhythmogenic mechanisms, but they have not been conventionally obtained from transgenic mice harboring mutations associated with inherited arrhythmic syndromes. In myocardial slices, CMs maintain their physiologic cell-to-cell connectivity while offering the practical advantages of a bidimensional preparation, and they are, therefore, well suited for fluorescence Ca^2+^ imaging. The use of multiphoton microscopy allowed imaging cells in the layers underneath the slice surface, thus leading to superficial CMs being inevitably damaged by the cutting procedure. CMs inside the slice volume responded to electrical stimulation with a synchronized, transient increase in cytoplasmic Ca^2+^ that was qualitatively comparable to that observed in isolated cells. Moreover, the kinetics of electrically stimulated Ca^2+^ changes was similar between WT and CPVT CMs in the slice, with the only difference observed being a moderate increase in the transient decay time, which might reflect the increased diastolic Ca^2+^ leak, as previously reported for single isolated CPVT CMs [14]. Exercise and adrenergic stress are well known arrhythmia triggers in CPVT, and NE stimulation, in experiments on isolated cells, caused increased spontaneous Ca^2+^ release and intracellular traveling Ca^2+^ waves during diastole. As expected, β-AR stimulation of our slices increased the gain of excitation–contraction coupling; in particular, we report that NE administration increases the amplitude and decreases the decay time of Ca^2+^ transients in both CPVT and control heart slices.

In perfect agreement with data obtained in isolated CMs, diastolic Ca^2+^ releases, during 1 Hz pacing, were observed with higher frequency in CPVT myocardial myocytes upon β-AR stimulation, whereas they were practically absent in the control conditions. Moreover, heart slices obtained from CPVT hearts exhibited a pro-arrhythmogenic substrate related to the development of Ca^2+^ *alternans* during β-AR stimulation. As reported by Weiss et al. [39], Ca^2+^ *alternans* at the cellular level play a key role in enhancing arrhythmic vulnerability, potentially causing focal ectopic triggers at the tissue level. In CPVT slices, we frequently monitored Ca^2+^ *alternans*, both concordant or discordant in amplitude among neighboring cells, a feature reported also by other groups [40]. It has been shown that Ca^2+^ alternans are a pathophysiologic mechanism of arrhythmia triggering [41], and their development may depend on an abnormal control of Ca^2+^ release from the SR, as well as on altered RyR2 refractoriness, as proposed by [42]. While, at the single-cell level, the occurrence of DAD eliciting an action potential is likely decreased by the reduction in SR [Ca^2+^] load, mechanisms not yet uncovered may promote spatial (i.e., in a group of neighboring CMs) and temporal synchronization throughout several cells in the tissue to act as effective arrhythmia triggers. Although the previous study by Cerrone and colleagues [34] identified the source of arrhythmic trigger in CPVT Purkinje fibers, CMs are capable of generating spontaneous Ca^2+^ release, DADs, and spontaneous APs in response to catecholaminergic stimulation, which makes them equally suited candidates to initiate ventricular arrhythmias in CPVT hearts [34,43].

We previously demonstrated that sympathetic neurons activate CM β-ARs by releasing NE in a restricted signaling domain confined within the limits of the structured contact site between neuronal varicosity and the coupled CM membrane [44]. In addition, 3D reconstruction of the SN network, in tissue clarified murine hearts, showed heterogeneous innervation [45,46], implying that CMs may receive heterogenous activation of β-ARs, enhancing SR Ca^2+^ load-driven diastolic leak and DAD generation in localized clusters of cells. It is tempting to speculate that the sympathetic network topology may add to the local mechanisms synchronizing triggered activity within a specific myocardial region. 

Hence, our finding adds to the existing literature the concept that regenerative SCRs in a single CM may directly and adversely engage the surrounding electrically excitable cells. In the heart slices, we observed that, in a large number of cases, Ca^2+^ waves in a CM were followed within a short time period by Ca^2+^ waves in neighboring cells. The temporal distance between these events usually fell under a “coupling” interval equal to the mean duration of a traveling wave (around 900 ms). Ca^2+^ waves can readily travel through the whole cell; however, in multicellular preparations, they generally do not easily propagate through gap junctions from one cell to the next one [30,47], excluding intercellular Ca^2+^ diffusion as the main cause of SCRs synchronization. Another study on papillary muscles from a CPVT-associated *CASQ2* mutated mouse model suggested the decreased RyR2 refractoriness as a determinant of synchronization between neighboring CMs [32]. Similarly, inhibition of RyR2 phosphorylation, via transduction of a CaMKII inhibiting peptide, reduced arrhythmias in a *RyR2*-linked CPVT model [48].

Although apparently in contrast with the “source–sink” model for electrotonically coupled CMs, our observation can be explained by considering that, when “source” CMs develop Ca^2+^-activated DADs, the depolarizing current would spread through the whole myocardium if not sufficiently sunk by the surrounding CMs. The current sink is paid with the slight, below-threshold depolarization, which increases SR Ca^2+^ release and facilitates cell-wide slow Ca^2+^ wave propagation [37]. The combined effect of these events may, therefore, increase the probability that the sink regenerates a source, thus determining a mechanism that leads to the recruitment and synchronization of DADs in larger groups of contiguous CMs (Figure 6).

## 5. Conclusions

The results of the current work show that, in neighboring CMs from KI mice harboring a CPVT-linked *RyR2* patient mutation, associated with increased channel leakiness, arrhythmogenic Ca^2+^ waves occur from self-amplifying alterations in local control of Ca^2+^ dynamics. The same behavior is appreciated in the multicellular myocardial slice model, in which complex multifactorial mechanisms are poised to increase arrhythmogenic Ca^2+^ wave risk.

## 6. Note

Some of the results presented in the manuscript stem from the PhD thesis of Dr Giulia Borile. 

## Data Availability

Data available upon request.

## Supplementary Materials

The following are available online at https://www.mdpi.com/article/10.3390/jcm10132821/s1: Supplementary data include supplementary text (supplementary methods; figure legends and references); Figure S1. Assessment of SERCA content in mouse hearts and Ca^2+^ decay time during postnatal development; Figure S2. Histological and immunofluorescence characterization of murine CPVT heart slices; Figure S3. Assessment of cell viability in murine CPVT heart slices; Figure S4. Loading of CPVT heart slices with the Ca^2+^ dye Fluo-4.
