# Supplementary material for: Multiphoton Imaging of Ca2+ Instability in Acute Myocardial Slices from a RyR2R2474S Murine Model of Catecholaminergic Polymorphic Ventricular Tachycardia
Source: J Clin Med. 2021 Jun 26;10(13):2821. doi: 10.3390/jcm10132821 (PMC8269190; doi:10.3390/jcm10132821)
Supplement: Supplementary file 1 [file jcm-10-02821-s001.zip › jcm-1243875-supplementary.pdf]

Journal of Clinical Medicine

MDPI

Supplementary content:

- Supplementary Methods;
- Supplementary Figures (1–4);
- Supplementary Figure Legends (1–4);
- Supplementary references.

Supplementary Methods

SM. 1 Histological analysis of heart slices
Acute ventricular slices, not used for imaging experiments, were fixed in 1% paraformaldehyde (PFA; *w/v* in PBS) for 15 min at room temperature. Slices were then included in freezing medium (OCT) and frozen in liquid nitrogen. Ten micrometer thick cryosections were cut with a cryostat (Leica Microsystems), as described before [1,2]. Cryosections underwent hematoxylin/eosin staining, using the Bio-Optica kit, following the manufacturer's instructions.

SM. 2. Immunofluorescence analysis of heart slices
A subset of heart cryosections described in *SM.1* were used for immunofluorescence analysis, which was performed following the protocol described in [1,2]. Sections were stained with an antibody to cardiac Troponin I (1:200) [3], while nuclei were counterstained with DAPI (1:5000). Sections were analyzed using the fluorescence microscope *Leica* DM6B, equipped with the CDD camera DFC300FX and the IM1000 software.

SM. 3. Assessment of cell viability on heart slices
To test cell viability, slices were loaded with propidium iodide (PI, Sigma-Aldrich) 0.5 μM in Tyrode solution for 30 min at 4 °C. Slices were then fixed with 1% PFA for 2 h at room temperature. Ten micrometer thick cryosections were cut with a cryostat (Leica Microsystems), and sections were analyzed using the fluorescence microscope *Leica* DM6B, equipped with the CDD camera DFC300FX and the IM1000 software.

SM. 4 Western blotting
Hearts from WT mice, at different postnatal stages (P2, P4, P10, and P21) were frozen in liquid nitrogen, immediately after removal from sacrificed animals. Proteins were extracted from tissue fragments following the protocol previously described in [1,2].

Supplementary Figure Legends

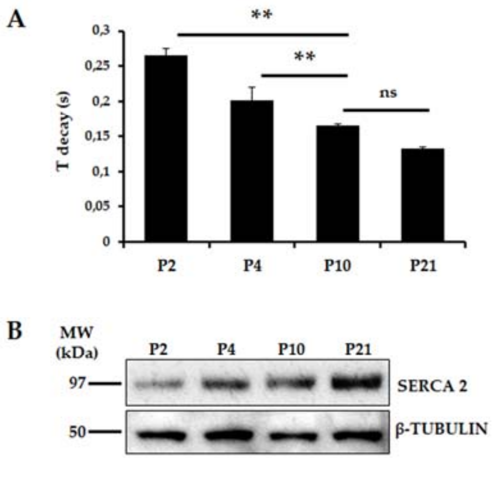

Figure S1. *Assessment of Ca^{2+} decay time and SERCA content in mouse hearts during postnatal development.*
(**a**) Decay time of Ca^{2+} transients in WT heart slices obtained at different ages, analyzed with confocal microscopy as described in Section 2. Bars represent SD; $n = 6$ hearts for each time points (** $p < 0.01$; ns = not significant). (**b**) Western blotting on heart extracts from normal mice at different postnatal stages (P2, P4, P10, and P21). β-Tubulin was used to ensure equal protein loading. MW, molecular weight.

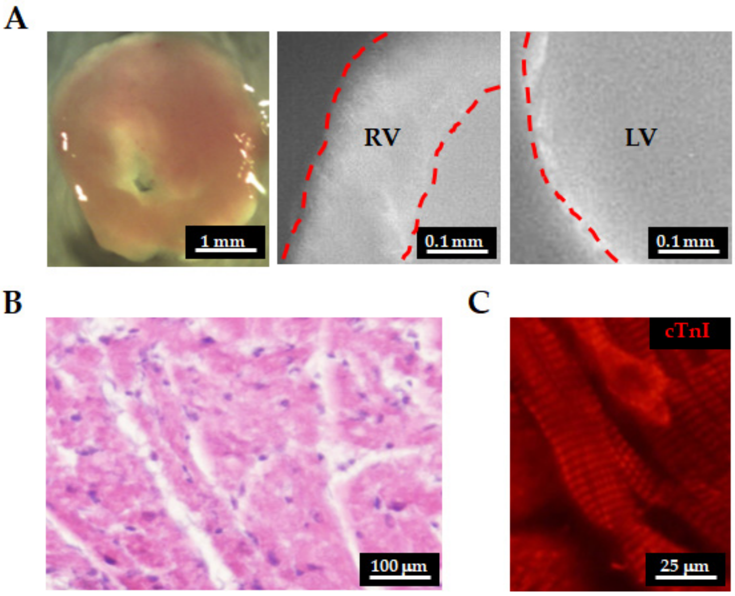

Figure S2. *Histological and immunofluorescence characterization of murine CPVT heart slices.*
(**a**) Stereomicroscope image of an acute heart slice (left panel) and two areas imaged in bright field focused on the right (middle panel) and left ventricle (right panel) walls, enclosed by the red dashed lines. (**b**) Hematoxylin–eosin staining on a thin cryosection obtained from a thick heart slice. (**c**) Immunofluorescence analysis of a thin cryosection, obtained from a thick heart slice (see **a**), stained with an antibody to cardiac Troponin-I (cTnI) [3].

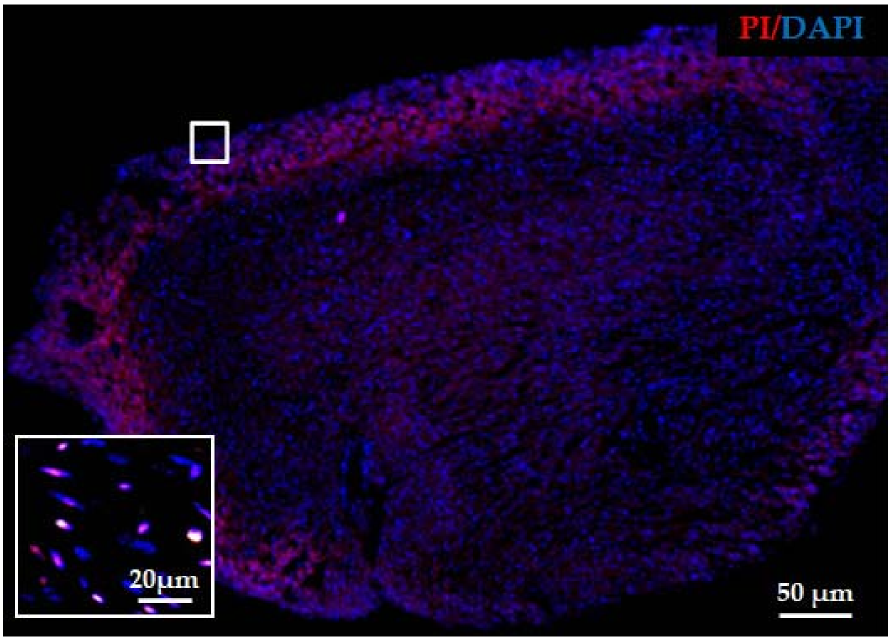

Figure S3. *Assessment of cell viability in murine CPVT heart slices.*
Transversal cryosection from a heart slice loaded with propidium iodide (PI, red signal) and co-stained with DAPI (blue signal). Sections were analyzed at the fluorescence microscope. The inset shows the high magnification of the white box, localized in the slice border, and evidences colocalization of PI and DAPI signals.

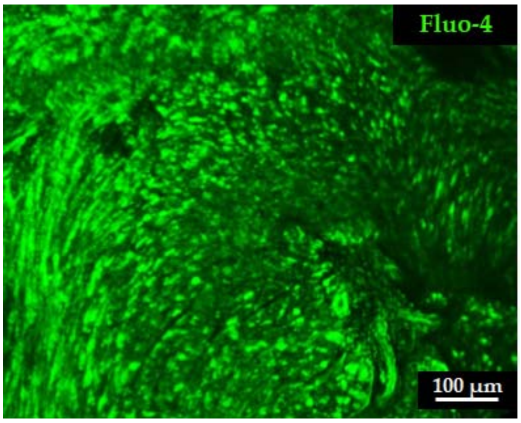

Figure S4. Loading of CPVT heart slices with the Ca^{2+} dye Fluo-4.
Fluorescence microscope image of a thick heart slice loaded with Fluo-4 (5 µM). Image is a detailed view of the right ventricle.

Supplementary References

1. Zaglia, T.; Milan, G.; Franzoso, M.; Bertaggia, E.; Pianca, N.; Piasentini, E.; Voltarelli, V.A.; Chiavegato, D.; Brum, P.C.; Glass, D.J.; Schiaffino, S.; Sandri, M.; Mongillo, M. Cardiac sympathetic neurons provide trophic signal to the heart via β2-adrenoceptor-dependent regulation of proteolysis. *Cardiovasc Res*. 2013, *97*(2), 240-50. https://doi.org/10.1093/cvr/cvs320.
2. Zaglia, T.; Milan, G.; Ruhs, A.; Franzoso, M.; Bertaggia, E.; Pianca, N.; Carpi, A.; Carullo, P.; Pesce, P.; Sacerdoti, D.; Sarais, C.; Catalucci, D.; Krüger, M.; Mongillo, M.; Sandri, M. Atrogin-1 deficiency promotes cardiomyopathy and premature death via impaired autophagy. *J Clin Invest*. 2014, *124*(6), 2410-24. https://doi.org/10.1172/JCI66339.
3. Saggin, L.; Gorza, L.; Ausoni, S.; Schiaffino, S. Troponin I switching in the developing heart. *J Biol Chem*. 1989, *264*(27), 16299-302.
